# Retraction: Paracrine interleukin-8 affects mesenchymal stem cells through the Akt pathway and enhances human umbilical vein endothelial cell proliferation and migration

**DOI:** 10.1042/BSR-2021-0198_RET

**Published:** 2021-12-17

**Keywords:** Bioinformatics, Human bone marrow mesenchymal stem cells, Human umbilical vein endothelial cells, Interleukin-8, Migration

This article is being retracted from *Bioscience Reports* at the request of the authors due to an image duplication in Figure 5G that the authors believe was introduced during the preparation of their figures. All authors believe that the integrity of the manuscript is therefore impaired, making it easy to erroneous understanding and wish to retract the article.

The Editor-in-Chief and Editorial Board agree with the retraction.

